# Two Cases of Peritonitis from Perforation Occurring in Typhoid Fever

**Published:** 1853-03

**Authors:** Austin W. Nichols

**Affiliations:** Jeff. Med. College


ART. IV.—*Two Cases of Peritonitis from Perforation occurring in Typhoid Fever.* Reported by AUSTIN W. NICHOLS.

PHILADELPHIA, Jan. 20, 1853.

DEAR DOCTOR:

The two following cases I have thought of sufficient interest to send you. If important enough to warrant an insertion in your Journal, they are at your disposal. They present the symptoms following perforation of the intestines during an attack of typhoid fever. In one of these, recovery took place; in the other, death terminated the disease.

I hope the class in the Buffalo University meets the expectations of its well-wishers. The Hospital presents advantages that I have not seen equaled in this city. I, for one, will return satisfied with the benefits to be derived from the teachings in the *Hospital of the Sisters of Charity.* With my best wishes for your prosperity, I remain,

Yours respectfully,

AUSTIN W. NICHOLS,

Jeff. Med. College.

CASE I. Typhoid fever. Peritonitis following perforation. Recovery.

A boy, aged 13 years, entered the Pennsylvania Hospital, Dr. Wood being in attendance. He had no aberration of mind; his countenance was dusky; tongue moist; skin hot and dry; pulse frequent, yet under 100; had diarrhœa; tympanites; gurgling; pain in right iliac region on pressure; no eruption; had epistaxis before his entrance; this was the first day he had taken to bed. The diagnosis was a mild attack of typhoid fever. The disease progressed favorably for ten days; there had been no necessity for medication with the exception of slight alcoholic stimulation; his diet was almost entirely of milk and farinaceous substances.

On the tenth day he was seized very suddenly with intolerable pain in the abdomen, shooting from the right side; his knees were drawn up and the cover was thrown off; excessive tenderness was found over the abdomen; his pulse was quick and small; his breathing costal and hurried; his countenance wore an anxious expression; his mind began to wander; these phenomena were sufficient to characterize a sudden attack of peritonitis.

The abdomen was immediately covered with a large blister, and this dressed with an emollient poultice. Morph. sulphat. gr. $\frac{1}{4}$ to $\frac{1}{3}$ was given every two hours, for two or three days; so that from three to four grains were administered in the twenty-four hours. At the same time, Mass. Pilul. Hyd. gr. j, was given every hour till some sensible improvement had taken place. His diet was restricted to arrow-root and milk. On the second day he began to grow better; and the inflammation subsided rapidly toward evening. For two or three days, he continued to amend, but his tongue was dry and his skin hot. Ol. Terebinth. gtts. x, ter die, was prescribed. From this time, he had no unfavorable symptoms; his appetite returned and gradually his strength. In a few weeks, he was about the ward; and now is perfectly well.

CASE II. Typhoid fever. Perforation. Death.

Girl, aged 9 years; began to complain on Tuesday of feeling ill. On the day previous, she was visited by her friends, who brought her cake and candy, of which she partook freely; some mild cathartic was administered, and the same medicine given on the next day, as the indisposition continued. The child took to bed on Saturday; and a tendency to sleep, and inattention to objects around her, was shown on this day. On Sunday evening, Dr. West called; the somnolency was prominent, sudamina on the neck were apparent, but there were no other symptoms to lead to a perfect diagnosis; there was a little fever, but no epistaxis, no diarrhœa, no tympanites, and no signs of an eruption. The Dr. supposed it to be an incipient case of typhoid fever. Some slight medication was employed, and the diet was ordered to be of milk and farinaceous substances. On Thursday there was a decided improvement in all the symptoms excepting the continuance of the somnolency. On Friday, the characteristic eruption of typhoid fever presented itself. On Sunday, sudamina were again observed; the somnolency still continued; the child had but little medicine, some mild stimulant was given, and the above diet was continued. In the afternoon, delirium of a most violent nature set in; the patient had to be held in bed, and restrained from injuring herself. She continued in this condition till early on Monday morning, when she expired. During this state, leeches were applied to the temples, blister to nape of the neck, and large doses of morphia were given internally. The post-mortem examination revealed ulceration, very extensive, of Peyer's glands, enlargement of many high up in the ileum; and in one patch near the ileo-colic valve, a perforation was discovered, the peritoneum in the vicinity alone was inflamed; the ventricles of the brain contained a little excess of fluid, otherwise the organ was healthy; there were no lesions in any other of the viscera.

